# The Lost Doctors

**DOI:** 10.12669/pjms.38.8.7022

**Published:** 2022

**Authors:** Aysha Zaheer

Medicine is considered the noblest of all professions. Globally, the number of females in the profession has increased in the past few decades.^1^ In Pakistan women had limited seats in public sector medical colleges in 1960s. Open merit was announced in 1980s which was reverted to limited number of seats for a few years. The decision was challenged in court and open merit was announced again. Percentage of females kept increasing from almost 50% up to year 2000. In the past decade this percentage has risen to 70% and above in most of the institutions.

According to a report published in 2006 the shortfall of doctors was expected to be anywhere from 58,000 to 451,000 in 2020 in Pakistan.^2^ Number of registered lady doctors is less as compared to the number who have graduated. There is a lost workforce.^3^ Is the open merit policy contributing to this? What are the other factors that might be responsible for this shortage of doctors? Let us analyze these questions in the light of research already conducted.

Since there is an open merit policy for admission in medical colleges, more and more females join the medical profession. Many common myths that are reverberated every now and then through laymen, institutions, media and social media. We need to have a look at them and the reality (published as research) (Table-I).

**Table-I:** Myths, Realities and Reality checks of professional life of female doctors.

Sr. no.	Myth	Reality	Interpretation/reality check
1	Females are occupying majority of seats in medical colleges although not capable to continue the line	Females compete for the seats and are selected on merit. (A merit list is announced each year)	Why do males fail to compete at induction level ?
2	Females join medicine to score high in the marriage market	It is still considered the noblest profession of all and therefore preferred by men and women alike. Parents encourage their daughters to become doctors.^1^	What is the impact on male marriage market?
3	Women lack in clinical skills and patient management	Patient management (30-day mortality and re-admission) is 4% better if managed by females. They are ethical and more likely to stick to best practices.^4^	Patient outcome is better when treated by female physicians
4	Women don’t give time properly to their workplace	They are dedicated workers but have to maintain a higher work-home life balance. They prefer their work to be planned ahead of time.^5^	Most women have a full responsibility towards home after work
5	Don’t excel in professional life	They do, in their preferred fields where they have autonomy. They are excellent administrators too	Certain specialties are preferred by women.^6^
6	Marriage is the end of a lady doctor’s career	Maximum number of serving lady doctors in our set up are married. They consider family, spouse as the biggest support in their professional life.^7^,^8^	Marriage provides a support system and is a source of emotional strength too.
7	Lady doctors quit professional life	True. As presented by PMDC registration data. Registered clinicians are almost equal in number although females are in majority at the time of admission^3^	This data does not indicate how many are serving in Pakistan

Shortage of doctors as per requirement has many reasons. Migration to other countries is quite common. United States and Ireland are the prime attractions. This migration is almost equally wanted in both genders (undergraduates).^9^

Medical training requires rigorous training. Working hours may exceed 80 hours per week. Doctors’ expectations are also high in terms of respect, salary, and recognition, which are barely met. Establishing a career abroad is sufficiently advocated and pursued by females. Common “push factors” being work place harassment, politics and low salary. The “pull factors” being better training and good salary.^2^ A survey with 366 immigrants in Ireland had highest number of doctors from Pakistan. The top most reasons for migration were to obtain postgraduate qualification, higher salary and family reasons. Once professional strength is achieved, they never want to return.^10^

Women leave medicine due to many reasons. The factors that lead to waste of valuable asset must be investigated and some remedial actions taken. Working long hours detached from the family is not acceptable to most if not all. Especially if they have to look after family members and children. The situation is quite similar in countries that have similar social and moral values. Preference for certain specialties is due to socially comfortable environment (GYNAE/OBS and pediatrics), better time management (basic sciences) although it may not be a specialty of choice.^1^,^6^ Career of lady doctors is affected by cultural as well as social norms. In some reasons may be personal (lack of passion or resilience). Professionally they may remain deficient if there is lack of professional coaching and mentoring.^7^

BMJ predicted this situation in 2009. It was suggested that the policies should be revised keeping an eye on the change in workforce trends. Specialty selection and gender choice should be kept in mind so that the patient care remains unaffected.^11^

Healthcare institutions must be aware of the difficulties faced by lady doctors at the beginning of their career. Job is described as and satisfaction is associated to male norms. Being a successful professional is not easy. Women need to develop the skills at the right time to excel. Government should review policies to protect female doctors from stressful situations at work which do not contribute to productivity. Female norms in a job should be determined. These include flexible working hours, provision of safe transport facilities, helping them in case of minor issues at home and neat clean day care centers. Open minded mentors should be appointed to help them with professional growth.^7^ Above all, family friendly policies should be introduced which will pay rich dividends.^5^

The number of seats for females entering in the medical institutions can be reviewed if the requirements per specialty is determined. Quota can be fixed accordingly.

## Note:

This was a special assignment during CME at UHS Lahore.
